# Dietary acrylamide intake and risk of breast cancer in the UK women's cohort

**DOI:** 10.1038/sj.bjc.6605956

**Published:** 2010-10-19

**Authors:** V J Burley, D C Greenwood, S J Hepworth, L K Fraser, T M de Kok, S G van Breda, S A Kyrtopoulos, M Botsivali, J Kleinjans, P A McKinney, J E Cade

**Affiliations:** 1 Centre for Epidemiology and Biostatistics, University of Leeds, Leeds LS2 9JT, UK; 2 Department of Health Risk Analysis and Toxicology, University of Maastricht, Maastricht, The Netherlands; 3 Institute of Biological Research and Biotechnology, National Hellenic Research Foundation, Athens, Greece

**Keywords:** cohort study, acrylamide, diet, breast cancer

## Abstract

**Background:**

No studies to date have demonstrated a clear association with breast cancer risk and dietary exposure to acrylamide.

**Methods:**

A 217-item food frequency questionnaire was used to estimate dietary acrylamide intake in 33 731 women aged 35–69 years from the UK Women's Cohort Study followed up for a median of 11 years.

**Results:**

In all, 1084 incident breast cancers occurred during follow-up. There was no evidence of an overall association between acrylamide intake and breast cancer (hazard ratio=1.08 per 10 *μ*g day^−1^, 95% CI: 0.98–1.18, *P*_trend_=0.1). There was a suggestion of a possible weak positive association between dietary acrylamide intake and premenopausal breast cancer after adjustment for potential confounders (hazard ratio=1.2, 95% CI: 1.0–1.3, *P*_trend_=0.008). There was no suggestion of any association for postmenopausal breast cancer (hazard ratio=1.0, 95% CI: 0.9–1.1, *P*_trend_=0.99).

**Conclusions:**

There is no evidence of an association between dietary acrylamide intake and breast cancer. A weak association may exist with premenopausal breast cancer, but requires further investigation.

Acrylamide is formed principally by the Maillard reaction between the amino acid asparagine and reducing sugars such as glucose (Stadler *et al*, 2002). Until its discovery in high temperature cooked carbohydrate-rich foods in 2002, it was thought that the main route of human exposure was occupational or from smoking (Smith *et al*, 2001). The finding of significant amounts of acrylamide in commonly consumed foods, such as potato crisps, French fries, coffee, biscuits and cold breakfast cereal (Tareke *et al*, 2002), raised health concerns in light of its classification as a probable human carcinogen by IARC (IARC Working Group on the Evaluation of Carcinogenic Risks to Humans, 1994). Animal studies have demonstrated that exposure to acrylamide causes a higher incidence of several tumours, including those of the mammary gland, but at levels of intake much greater than the equivalent typically observed in humans (Besaratinia and Pfeifer, 2003; Friedman, 2003; Rice, 2005; Spivey, 2010; Tardiff *et al*, 2010). As a consequence, several studies have attempted to assess whether acrylamide exposure through foods increases cancer risk in humans.

Some evidence has been found of an association between dietary acrylamide and endometrial and ovarian cancers (Hogervorst *et al*, 2007), but no studies to date have demonstrated a clear association with breast cancer risk and dietary exposure to acrylamide (Mucci *et al*, 2005; Pelucchi *et al*, 2006; Hogervorst *et al*, 2007; Larsson *et al*, 2009; Wilson *et al*, 2009a), apart from one study suggesting a possible positive association with breast cancer among a subgroup of postmenopausal never-smokers (Pedersen *et al*, 2010). In addition, a recent biomarker study found a possible association with acrylamide–haemoglobin adduct levels for oestrogen receptor positive postmenopausal breast cancers, though there was no overall association for postmenopausal breast cancers, and no investigation of premenopausal breast cancers.

We therefore aimed to investigate the association between dietary acrylamide intake and all breast cancers in a large prospective cohort of women. The UK Women's Cohort Study (UKWCS) is participating in the NewGeneris consortium investigating dietary exposures to potentially carcinogenic compounds (Merlo *et al*, 2009), and is therefore ideally suited to investigating this question.

## Materials and Methods

The UKWCS is formed from a sample of 35 372 women who completed a 217-item food frequency questionnaire (FFQ) during 1995–1998 (Cade *et al*, 2004a). The FFQ was based on the FFQ used in the UK arm of the European Prospective Investigation into Cancer study, which had been validated against 16-day weighed records and biomarkers (Bingham *et al*, 1997), and adapted for this population in line with best practise (Cade *et al*, 2004b) followed by further validation (Calvert *et al*, 1997; Buckley *et al*, 2001; Spence *et al*, 2002). Subjects in the UKWCS are mainly white, affluent, well-educated women, and were initially aged between 35 and 69 years. They are generally health conscious with only 11% current smokers and 58% taking dietary supplements (Cade *et al*, 2004a). Physical activity levels and self-reported weight, height and waist and hip circumferences were also collected at baseline. Ethical approval was obtained from 174 local research ethics committees as described previously (Cade *et al*, 2007; Taylor *et al*, 2007). The cohort was designed to include a wide range of dietary patterns with similar large numbers of women consuming a vegetarian type diet, women who ate fish but not meat and meat eaters, to achieve adequate power while minimising the effects of measurement error (White *et al*, 1994; Kaaks and Riboli, 1997; Schatzkin *et al*, 2001).

Estimated intakes of acrylamide were calculated for women participating in the UKWCS using the frequencies of consumption of 24 food items reported in the FFQ that were potential sources of dietary acrylamide, broadly grouped into potato chips, crisp breads, breakfast cereals, pastries and bakery products, coffee, bread, biscuits and potato crisps. Toast was not included as a separate item in the FFQ, but was included as bread. Estimated acrylamide content was based on standard portion sizes (Food Standards Agency, 2002) and European Union (EU) estimates of acrylamide content of foods (European Commission Institute for Reference Materials and Measurements, 2006). The EU database was set up to permit monitoring of acrylamide levels in food products throughout the whole EU, which includes the UK. A recent validation study compared estimated acrylamide intake based on average acrylamide levels of food items, such as those available in the EU database, compared with chemically analysed content (Konings *et al*, 2010). The correlation between chemically determined acrylamide content and estimated acrylamide content was very high, indicating that using single acrylamide values for individual foods based on average values of several available samples, such as that reported in the EU database, results in a good rank ordering of most subjects, despite the large variation of acrylamide concentrations within single foods. We further adapted the database for the UK population by using coffee strengths and portion sizes based on those appropriate for the UK (Food Standards Agency, 2002), and in addition we assumed that, for exposure estimates in this population of middle-aged women, any potato chips consumed were generally broader than the narrower French fries, and so with a relatively lower acrylamide content. The FFQ was repeated in a random subsample of 1859 women 5 years after completion of the first questionnaire. Spearman's correlation was 0.61 for total dietary acrylamide intake, with similarly high values across all dietary sources of acrylamide.

Study participants were registered with the National Health Service Central Register so that all incident cancer cases and deaths were notified to the study team. Incident cancers and cause of death were coded according to the International Classification of Diseases 9 and 10. An incident breast cancer was taken to be malignant breast cancer (ICD9 174, ICD10 C50) occurring after completion of the FFQ. Breast cancer cases’ survival time was taken as date of completing the FFQ until date of diagnosis. Non-cases’ survival was taken as being from date of enrolment until death or end of follow-up (1 January 2008) whichever was the first. Menopausal status was coded using specific criteria related to menstrual and obstetric history and age.

### Statistical analysis

Cox's proportional hazards regression was used to explore the relationship between estimated dietary acrylamide intakes and risk of breast cancer using Stata version 11 (StataCorp., 2009). Women were excluded if they had extremely high (>6000 kcal day^−1^) or low (<500 kcal day^−1^) reported total energy intake, as were women with prevalent breast cancer. Associations were estimated for premenopausal and postmenopausal women separately and combined, first as a simple model adjusting for age, smoking status and amount smoked (model 1), and second as a full model additionally adjusting for weight, height, physical activity (hours per day sufficiently vigorous to cause sweating), oral contraceptive use, hormone replacement therapy use, parity, age at menarche, alcohol intake (as grams of ethanol per day), energy intake other than from alcohol, and level of education (model 2). Tests for trend were based on fitting the linear trend over the continuous measure of exposure.

To assess sensitivity of results to undiagnosed prevalent breast cancers at the time the FFQ was completed, analyses were repeated after excluding any events occurring within 6 months of completing the FFQ. A further analysis excluding current or ever smokers was undertaken as exposure to acrylamide through tobacco smoke inhalation is likely to be a more important source of exposure than dietary sources alone (Olesen *et al*, 2008). For this subgroup there was no need to adjust for smoking habits in the models. To investigate the sensitivity of results to including all bread as untoasted, we assumed half of bread was toasted, with associated higher acrylamide content, and re-ran all analyses.

## Results

The median follow up was for 11 years, with 1084 incident breast cancers recorded. Using information collected at baseline, 15 951 women were classified as being premenopausal and 17 779 postmenopausal. Demographic and clinical characteristics of the study participants are shown in Table 1 according to fifth of acrylamide intake. The women with the highest dietary acrylamide intakes were slightly younger, with higher body mass index, less physically active, less educated, more likely to have children, have a higher total energy intake, have a lower alcohol intake, but more likely to smoke.

Median dietary acrylamide intake was 15 *μ*g day^−1^ (0.23 *μ*g kg^−1^ day^−1^), with an inter-quartile range of 10–21 *μ*g day^−1^ (0.15–0.33 *μ*g kg^−1^ day^−1^). Only 316 out of 33 061 (1%) of women consumed more than 50 *μ*g day^−1^ of dietary acrylamide. Only 99 out of 33 061 (0.3%) of participants consumed in excess of 1 *μ*g kg^−1^ day^−1^, a threshold of intake cited by the World Health Organization in risk assessment models (Joint Food and Agriculture Organization of the United Nations/World Health Organization consultation on health implications of acrylamide in food, 2002), and still fewer exceed other estimates of tolerable daily intake (Tardiff *et al*, 2010). The sources with greatest contribution to total dietary acrylamide intake in these middle-aged women in the United Kingdom were potato chips (28% of intake), bakery goods (17%), potato crisps (14%), bread (10%), biscuits (9%) and coffee (8%).

Overall, there was little evidence of an association between dietary acrylamide intake and breast cancer when premenopausal and postmenopausal cancers were combined (hazard ratio=1.08 per 10 *μ*g day^−1^, 95% CI: 0.98–1.18, *P*_trend_=0.1) (Table 2). However, there was evidence of a positive dose-response association between dietary acrylamide intake and premenopausal breast cancer after adjustment for potential confounders. Women in the highest fifth of dietary acrylamide intake had ∼50% higher risk of subsequent breast cancer than those in the lowest fifth (hazard ratio=1.47, 95% CI: 0.96–2.27) with associated risk increasing by ∼20% with every 10 *μ*g day^−1^ increase in acrylamide intake (hazard ratio=1.18, 95% CI: 1.05–1.34, *P*_trend_=0.008). However, there was no such association for postmenopausal breast cancer (hazard ratio=1.00, 95% CI: 0.88–1.14, *P*_trend_=0.99), with similar numbers of premenopausal and postmenopausal women offering similar power for each comparison.

These results were unchanged by excluding women with breast cancers diagnosed within 6 months of completing the FFQ. When these analyses were further restricted to the subgroup of women who had never smoked, the associations between dietary acrylamide and breast cancer were less apparent, though power was much reduced (Table 3). If half of all recorded bread intake were as toast, this would increase the median acrylamide intake to 20 *μ*g day^−1^ (inter-quartile range 14–27 *μ*g day^−1^) or 0.31 *μ*g kg^−1^ day^−1^ (inter-quartile range 0.21–0.43 *μ*g kg^−1^ day^−1^). However, dose-response trends were very much the same as before.

## Discussion

In our large UK cohort, we have shown no association between dietary acrylamide intake and increased risk of breast cancer, either overall or for postmenopausal breast cancer, for modest intakes consumed as part of a usual diet (<50 *μ*g day^−1^). However, there was some evidence for a positive association with premenopausal breast cancer. There was a weak dose-response relationship, but the association was particularly apparent in the highest fifth of dietary intake (>23 *μ*g day^−1^).

Given that smoking is not a risk factor for breast cancer, and that smokers have substantially higher levels of haemoglobin adducts of acrylamide and glycidamide than non-smokers (Hagmar *et al*, 2005; Bjellaas *et al*, 2007), much higher than that associated with dietary intake, any association between dietary intake and breast cancer is surprising. Given also that the acrylamide content of tobacco outweighs any that of any dietary intake, it was also important to repeat the analyses amongst a subgroup of never-smokers.

The association was less apparent in the subgroup of premenopausal women, who had never smoked. It is possible that the lack of a clear association amongst the never-smokers could be explained by insufficient power to detect increased risk, as this subgroup comprises just a quarter of the cohort. However, the overall number of cases of breast cancer reported in our cohort are comparable to those in other major studies, such as the Nurses’ Health Study II (Wilson *et al*, 2009a), which found no associations between intakes of acrylamide, or foods potentially high in acrylamide, and invasive breast cancer.

The FFQ used in our cohort is similar to that used in the European Prospective Investigation of Cancer (Riboli and Kaaks, 1997) and follows recommendations for good design (Cade *et al*, 2004b). However, no distinction was made between bread and toasted bread, which would have higher acrylamide content. When the robustness of results was assessed by assuming a proportion of bread was toasted, conclusions were unchanged.

Our average intakes were at the lower end of those previously reported from other populations (Swiss Federal Office of Public Health, 2002; Dybing and Sanner, 2003; Svensson *et al*, 2003; US Food and Drug Administration, 2006; Larsson *et al*, 2009; Wilson *et al*, 2009b). Partly this is an artefact of presenting the more appropriate median intake, which is generally lower than the mean (Dybing *et al*, 2005), and partly underestimation caused by grouping bread and toast together. However, we believe these lower intakes also reflect the dietary habits of our middle-aged female UK population who, at the time of completing the FFQ, consumed relatively fewer potato chips and crisps, generally preferred tea rather than coffee, and consumed weaker coffee than some other countries.

Our intakes are closer to those found in the US, Switzerland and the Netherlands, than to those found in Scandinavia. For example, the proportion of acrylamide estimated to be from coffee in our population (8%) is in keeping with figures from the Netherlands and US (Mucci and Wilson, 2008), but very different from the 39% reported in Sweden. The UKWCS participants generally have a health conscious outlook with relatively low smoking rates and low body mass index (Cade *et al*, 2004a). It is therefore possible that less healthy dietary patterns were under-represented in our cohort. This may also have contributed to the relatively low daily intake of acrylamide in the UKWCS compared with other studies.

Even in our relatively health-conscious cohort of middle-aged women, the main sources of acrylamide were chips and crisps. It is therefore possible that any measure of dietary acrylamide intake may be a proxy for poor diet, which could predict breast cancer. This in turn may be predictive of increased risk of breast cancer through other dietary risk factors such as higher fat or processed meat intakes (Bingham *et al*, 2003; Cade *et al*, 2007; Taylor *et al*, 2007).

As this is a prospective study, recall bias is unlikely. However, it is possible that current estimates of acrylamide content in food are incomplete, which may have lead to some misclassification of exposure categories. In addition, estimates of acrylamide exposure were based on the responses to one FFQ administered at recruitment into the cohort. As the consumption of foods thought to be contaminated with acrylamide may have changed over time, it is possible that our estimates of exposure might have been improved with repeated dietary assessments. The range of acrylamide levels reported for specific products, with different methods of preparation, is wide, and even within brands considerable variation occurs between different batches (European Food Safety Authority, 2010). As the FFQ does not request brand-specific information, and individuals do not necessarily remain loyal to one brand, this variation could not be accounted for in the exposure calculations. In particular, the acrylamide content of coffee will differ by brand and strength of coffee consumed (Bagdonaite *et al*, 2008). This choice varies considerably between individuals, and across different countries. However, studies have shown that it is possible for FFQs to estimate dietary acrylamide intake well (Dybing *et al*, 2005; Brantsaeter *et al*, 2008).

Table 4 briefly summaries the results from some existing studies of dietary acrylamide and breast cancer. The majority of studies, like ours, found no evidence of an association between dietary acrylamide intake and incident breast cancer, at least within the range of intakes studied. The Netherlands Cohort study only found indications of a positive association between dietary acrylamide intake and a subgroup of hormone receptor-positive breast cancers in postmenopausal women, who have never smoked (Hogervorst *et al*, 2007; Pedersen *et al*, 2010), and recognised that further studies were required to confirm or refute this. However, there is other evidence that acrylamide may interfere with hormone systems (Hogervorst *et al*, 2010). A weakness of our study was that we did not have information on hormone receptor status and so we were unable to selectively report results by tumour receptor positivity.

The strongest suggestion of a positive association comes from a large prospective cohort (Olesen *et al*, 2008), where the association between breast cancer and biomarkers for acrylamide exposure was assessed using haemoglobin adduct levels, as well as glycidamide, which is the genotoxic metabolite of acrylamide. After adjustment for smoking behaviour in their nested case–control study, a positive association was seen between acrylamide and haemoglobin adduct levels and oestrogen receptor positive breast cancer. Our study takes this further and relates breast cancer directly to the original dietary consumption that may contribute to the adduct levels and metabolite concentrations. A recent cross-sectional analysis of individuals found higher biomarker values of acrylamide in the United Kingdom than in other European countries (Vesper *et al*, 2008), suggesting that this may be of particular importance in the United Kingdom, and a possible explanation for any differences between our results and those from other countries.

There is some mechanistic evidence to support this epidemiological evidence. In animal studies, acrylamide is causally associated with mammary tumour development (Johnson *et al*, 1986; Friedman *et al*, 1995; Rice, 2005; Wilson *et al*, 2009a), but to date there has been less evidence that acrylamide consumption at levels typically observed in normal diets is related to human breast cancer risk. The typical acrylamide intake of women in our study is substantially lower than the dose levels that have been found to cause effects in these animal studies.

Given the widespread presence of acrylamide in food and the relatively common nature of breast cancer, even a small association between the two would have important public health implications. In this first UK study, no overall association between dietary acrylamide intake and breast cancer risk was found, though a weak positive association was observed among premenopausal breast cancers, but further investigation is needed.

## Figures and Tables

**Table 1:** Demographic and clinical characteristics of women in the UKWCS by fifth of intake of acrylamide

	**Acrylamide intake**				
**Fifth**	**1**	**2**	**3**	**4**	**5**
**Mean (*μ*g day^−1^)**	**6**	**11**	**15**	**20**	**32**
**Range (*μ*g day^−1^)**	**0–9**	**9–13**	**13–17**	**17–23**	**23–150**
Mean age (years) (s.d.)	52.8 (9.2)	52.7 (9.1)	52.5 (9.3)	51.6 (9.3)	51.4 (9.5)
Mean BMI (kg m^−2^) (s.d.)	24.0 (4.2)	24.5 (4.4)	24.4 (4.1)	24.6 (4.6)	24.8 (4.5)
Mean physical activity (hours per day) (s.d.)	0.3 (0.5)	0.3 (0.5)	0.3 (0.5)	0.2 (0.4)	0.2 (0.5)
Mean age at menarche (years) (s.d.)	12.8 (1.7)	12.8 (1.6)	12.8 (1.6)	12.8 (1.6)	12.9 (1.6)
Current HRT use (%)	1336 (20%)	1386 (21%)	1348 (20%)	1239 (19%)	1206 (18%)
Current OCP use (%)	251 (4%)	232 (3%)	278 (4%)	264 (4%)	266 (4%)
Nulliparous (%)	1954 (29%)	1614 (24%)	1394 (21%)	1346 (20%)	1240 (18%)
Postmenopause (%)	3776 (56%)	3716 (55%)	3620 (54%)	3360 (50%)	3300 (49%)
Incident breast cancer (%)	220 (3%)	213 (3%)	219 (3%)	211 (3%)	218 (3%)
Mean total energy intake (MJ) (s.d.)	8.0 (2.4)	8.9 (2.4)	9.6 (2.4)	10.4 (2.6)	12.1 (3.3)
Mean ethanol intake (g day^−1^) (s.d.)	9.0 (11.4)	9.1 (10.5)	8.7 (10.6)	8.5 (10.1)	8.0 (10.1)
Current smoker (%)	684 (11%)	682 (10%)	710 (11%)	718 (11%)	846 (13%)
No education >14 years (%)	840 (14%)	951 (15%)	1038 (17%)	1076 (17%)	1291 (21%)

Abbreviations: BMI=body mass index; HRT=hormone replacement therapy; OCP=oral contraceptive pill; UKWCS=UK Women's Cohort Study.

**Table 2:** Hazard ratios of pre- and post-menopausal breast cancer according to acrylamide intake for all participants

			**Model 1** a			**Model 2** b		
**Fifth of acrylamide intake**	**Range (*μ*g day**^−1^)	**Cases/Total**	**HR**	**95% CI**	***P*_*trend*_** c	**HR**	**95% CI**	***P*_*trend*_** c
*All breast cancers*								
1	0–9	220/6747	1.00	—		1.00	—	
2	9–13	213/6748	0.92	(0.74, 1.13)		1.06	(0.83, 1.35)	
3	13–17	219/6747	0.90	(0.73, 1.11)		1.05	(0.82, 1.34)	
4	17–23	211/6748	0.92	(0.75, 1.14)		1.12	(0.87, 1.45)	
5	23–150	218/6748	0.97	(0.79, 1.19)		1.16	(0.88, 1.52)	
*Trend (per 10 μg day*^−*1*^)			*0.99*	*(0.92, 1.06)*	*0.7*	*1.08*	*(0.98, 1.18)*	*0.1*
								
*Premenopausal*								
1	0–9	73/2956	1.00	—		1.00	—	
2	9–13	85/3032	1.03	(0.7, 1.5)		1.06	(0.71, 1.59)	
3	13–17	88/3127	1.01	(0.7, 1.4)		1.15	(0.77, 1.71)	
4	17–23	90/3388	0.95	(0.7, 1.3)		1.15	(0.76, 1.73)	
5	23–150	102/3448	1.20	(0.9, 1.7)		1.47	(0.96, 2.27)	
*Trend (per 10 μg day*^−*1*^)			*1.06*	*(0.96, 1.17)*	*0.2*	*1.18*	*(1.05, 1.34)*	*0.008*
								
*Postmenopausal*								
1	0–9	135/3776	1.00	—		1.00	—	
2	9–13	128/3716	0.96	(0.7, 1.2)		1.06	(0.78, 1.44)	
3	13–17	131/3620	0.95	(0.7, 1.2)		1.00	(0.73, 1.38)	
4	17–23	121/3360	1.03	(0.8, 1.3)		1.14	(0.82, 1.58)	
5	23–150	116/3300	0.97	(0.7, 1.3)		0.97	(0.68, 1.39)	
*Trend (per 10 μg day*^−*1*^)			*0.98*	*(0.90, 1.07)*	*0.7*	*1.00*	*(0.88, 1.14)*	*0.99*

Abbreviations: CI=confidence interval; HR=hazard ratio.

a Model 1 is adjusted for age.

b Model 2 is also adjusted for smoking status and amount smoked weight, height, physical activity (hours per day sufficiently vigorous to cause sweating), oral contraceptive use, hormone replacement therapy use, parity, age at menarche, alcohol intake (as grams of ethanol per day), energy intake other than from alcohol, and level of education.

c *P*-value for trend is based on fitting the linear trend over the continuous exposure.

**Table 3:** Hazard ratios of pre- and post-menopausal breast cancer according to acrylamide intake for women who have never smoked

			**Model 1** a			**Model 2** b		
**Fifth of acrylamide intake**	**Range (*μ*g day^−1^)**	**Cases/Total**	**HR**	**95% CI**	***P*_*trend*_** c	**HR**	**95% CI**	***P*_*trend*_** c
*All breast cancers*								
1	0–9	128/3584	1.00	—		1.00	—	
2	9–13	114/3745	0.78	(0.60, 1.03)		0.87	(0.63, 1.20)	
3	13–17	130/3847	0.82	(0.63, 1.07)		0.95	(0.69, 1.30)	
4	17–23	120/3885	0.79	(0.60, 1.03)		0.96	(0.69, 1.34)	
5	23–150	115/3841	0.82	(0.62, 1.07)		0.98	(0.69, 1.40)	
*Trend (per 10 μg day*^−*1*^)			*0.94*	*(0.85, 1.04)*	*0.2*	*1.06*	*(0.93, 1.21)*	*0.4*
								
*Premenopausal*								
1	0–9	45/1604	1.00	—		1.00	—	
2	9–13	38/1709	0.73	(0.45, 1.19)		0.68	(0.39, 1.20)	
3	13–17	59/1805	1.04	(0.68, 1.61)		1.12	(0.69, 1.83)	
4	17–23	54/1990	0.80	(0.51, 1.25)		0.98	(0.59, 1.62)	
5	23–150	57/2024	1.00	(0.65, 1.54)		1.17	(0.69, 2.00)	
*Trend (per 10 μg day*^−*1*^)			*1.01*	*(0.88, 1.15)*	*0.9*	*1.13*	*(0.95, 1.33)*	*0.2*
								
*Postmenopausal*								
1	0–9	76/1972	1.00	—		1.00	—	
2	9–13	76/2036	0.89	(0.64, 1.26)		0.98	(0.66, 1.46)	
3	13–17	71/2042	0.79	(0.56, 1.13)		0.83	(0.54, 1.26)	
4	17–23	66/1895	0.89	(0.63, 1.25)		0.99	(0.64, 1.53)	
5	23–150	58/1817	0.81	(0.57, 1.16)		0.86	(0.53, 1.37)	
*Trend (per 10 μg day*^−*1*^)			*0.95*	*(0.83, 1.08)*	*0.4*	*1.01*	*(0.83, 1.24)*	*0.9*

Abbreviations: CI=confidence interval; HR=hazard ratio.

a Model 1 is adjusted for age.

b Model 2 is also adjusted for smoking status and amount smoked weight, height, physical activity (hours per day sufficiently vigorous to cause sweating), oral contraceptive use, hormone replacement therapy use, parity, age at menarche, alcohol intake (as grams of ethanol per day), energy intake other than from alcohol, and level of education.

c *P*-value for trend is based on fitting the linear trend over the continuous exposure.

**Table 4:** A brief summary of some existing prospective studies of dietary acrylamide and breast cancer incidence in humans

**Study**	**Population**	**Dietary acrylamide intake**	**Main findings RR (95% CI)**
Women's Lifestyle and Health Cohort (Mucci *et al*, 2005)	Cohort of 43 404 women in Sweden, mean age 39, 91% premenopausal, with 667 incident cases.	Measured by 98-item FFQ.	RR (highest : lowest fifth)=1.19 (0.91, 1.55)
The Italian multicentre case–control study of breast cancer (Pelucchi *et al*, 2006)	Hospital-based case–control study in Italy and Switzerland between 1991 and 2001 with 2900 cases and 3122 controls.	Measured by 78-item FFQ.	RR (highest : lowest fifth)=1.06 (0.88, 1.28)
The Danish Diet, Cancer and Health study (Olesen *et al*, 2008)	Case–control nested with a cohort of 24 697 postmenopausal women in Denmark, aged 50–64 years, with 374 incident cases.	Haemoglobin adducts of acrylamide.	Per 10-fold increase in acrylamide adducts, RR=1.05 (0.66, 1.69); RR=2.7 (1.1, 6.6) for ER+.
The Swedish Mammography Cohort (Larsson *et al*, 2009)	Cohort of 61 433 women attending mammography in Sweden, aged 42–72 years, with 2952 incident cases.	Measured by 67-item and 96-item FFQs.	RR (highest : lowest quarter)=0.91 (0.80, 1.02); 0.89 (95% CI: 0.74, 1.08) for ER+PR+ 1.17 (95% CI: 0.84, 1.64) for ER+PR-; 0.91 (95% CI: 0.61, 1.38) for ER-PR-.
Nurses’ Health Study II (Wilson *et al*, 2009a)	Cohort of 90 628 premenopausal women completing an FFQ in 1991, aged 27–44 years, with 1179 incident cases.	Measured by 130-item FFQ.	RR (highest : lowest fifth)=0.92 (0.76, 1.11); 1.31 (0.87, 1.97) for ER+ 1.47 (0.86, 2.51) for PR+ 1.43 (0.83, 2.46) for ER+PR+.
The Netherlands Cohort (Hogervorst *et al*, 2007; Pedersen *et al*, 2010)	Case–cohort analysis nested within a cohort of 62 573 postmenopausal women in the Netherlands, aged 55–69 years, with 2225 incident cases.	Measured by 150-item FFQ.	RR (highest : lowest fifth)=0.92 (0.73, 1.15)
The United Kingdom Women's Cohort study (Burley *et al*, 2010)	Cohort of 33 731 women in the United Kingdom, aged 35–69 years, 47% premenopausal, with 1084 incident cases.	Measured by 217-item FFQ.	RR (highest : lowest fifth)=1.16 (0.88, 1.52); 1.47 (0.96, 2.27) for premenopausal; 0.97 (0.88, 1.14) for postmenopausal.

Abbreviations: CI=confidence interval; ER+=estrogen receptor positive breast cancers; FFQ=food frequency questionnaire; PR+=progesterone receptor positive breast cancers; RR=relative risk.
